# A case study on the bearing characteristics of a bottom uplift pile in a layered foundation

**DOI:** 10.1038/s41598-022-27105-x

**Published:** 2022-12-28

**Authors:** Laping He, Xuwei Chen, Zhengzhen Wang, Yun Han, Tiantao Su, Guoliang Dai, Enxiang Zhang, Zhao Long

**Affiliations:** 1 Gansu CSCEC Municipal Engineering Investigation and Design Institute Co., Ltd., Lanzhou, 730000 Gansu China; 2 CSCEC AECOM Consultants Co., Ltd., Lanzhou, 730000 Gansu China; 3 School of Civil Engineering, Lanzhou University of Technology, Lanzhou, 730050 Gansu China; 4 Gansu Civil Aviation Airport Group Co., Ltd., Lanzhou, 730050 Gansu China; 5 School of Civil Engineering, Southeast University, Nanjing, 210000 Jiangsu China

**Keywords:** Environmental sciences, Civil engineering, Energy infrastructure

## Abstract

The bottom uplift pile, which has been applied in practical projects, has the following advantages: the pile body is not easy to crack, good bearing characteristics, and small displacement of the pile top. Based on the bearing capacity test of foundation piles in the third stage expansion project of Lanzhou Zhongchuan International Airport, the upper part pile of the self-balancing test method was used to simulate the bottom uplift pile, and the anchor piles in the anchor pile method were regarded as normal uplift piles. The bearing characteristics of the bottom uplift pile in a layered foundation were studied by comparing these two kinds of piles. The results show that under the same displacement of the pile top, the ultimate uplift bearing capacity of the bottom uplift pile can be more than twice that of the normal uplift pile because of the fully exerted frictional resistance of the soil at the bottom of the pile, the Poisson effect of the pile body and the avoidance of the influence of pile body deformation on the pile top displacement. The maximum axial force of the bottom uplift pile appears at the bottom of the pile and gradually decreases from the bottom to the top, which is opposite to that of the normal uplift pile. The properties and thickness of the soil layers around the pile have a great influence on the distribution curves of the frictional resistance along the pile length of the two kinds of uplift piles. With changing soil layer conditions, the distribution curve may be a "parabola", a "straight line" or a "double line". The soil property plays a decisive role in the frictional resistance, which may cause softening. The influence of the pile diameter on the ultimate uplift bearing capacity is greater than that of the pile length, while the elastic modulus of the pile has little influence.

## Introduction

**Figure 1:**
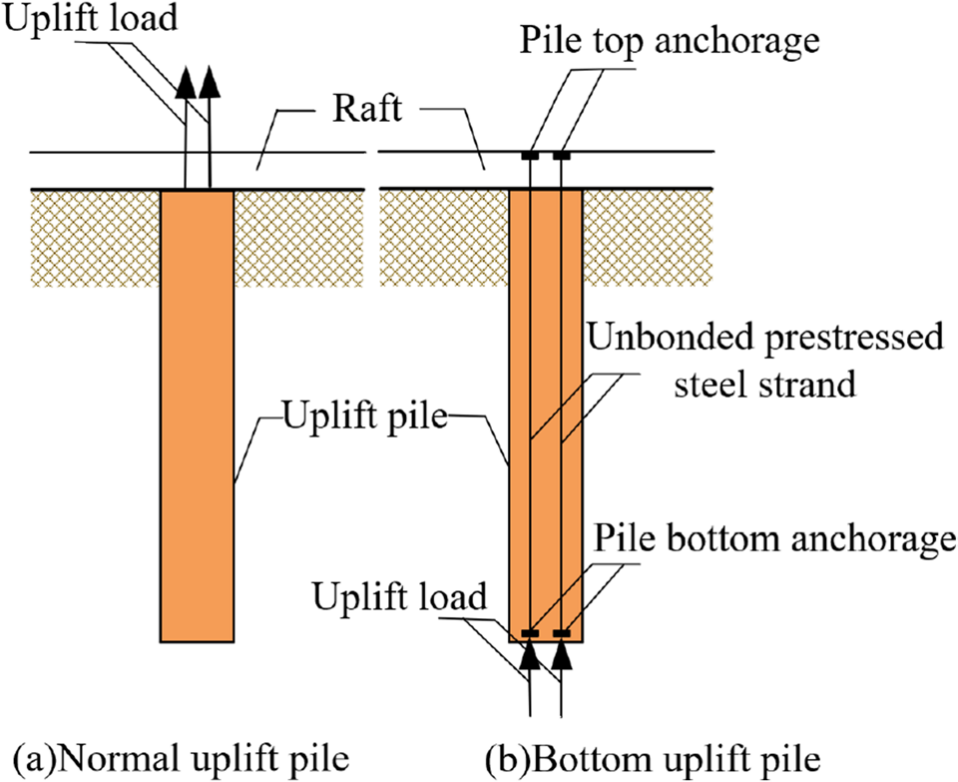
Schematic diagram of uplift piles.

With the attention given to groundwater resources, the anti-floating problem of buildings has become increasingly prominent. Uplift piles are one of the most widely used anti-floating foundations^1^. Compared with a normal uplift pile with tension at the top of the pile, bottom uplift piles have the following characteristics: the concrete of the pile shaft is not easy to crack, the deformation of the pile shaft has little influence on the displacement of the pile top, and the friction of the deep soil can be fully exerted. The force comparison diagram with a normal uplift pile is shown in Fig. 1^2,3^.

Many scholars have carried out in-depth research on normal uplift piles. Yang^4^ proposed a calculation method for the bearing capacity of uplift piles based on field measured data. Based on the failure surface of model tests, Hong et al.^5^ found an analytical approach to predict the uplift bearing capacity of a micropile. Lamparuthi et al.^6^ performed laboratory pullout tests and introduced the effective diameter and effective embedment ratio of uplift piles. Deshmukh et al.^7^ established an analysis method for uplift piles using Kotter’s equation. Wang et al.^8^ studied the development of the lateral friction of uplift piles in soft rock with shallow overburden through field tests. Basha et al.^9^ performed a series of model tests to study the influence of the groundwater level on the uplift bearing capacity of a single pile. Sakr et al.^10^ tried to use an anchor wing to improve the uplift capacity of a pile and found that this method is effective. Abdelgwad et al.^11^ conducted thirty-nine tests of enlarged base piles and normal piles to investigate the effect of enlarged bases. Based on the normal uplift pile, the bottom uplift pile has been invited, and an increasing number of researchers have tried to determine its bearing mechanism. Shao et al.^12^ discussed the influence of the Poisson effect on the bearing capacity of normal uplift and bottom uplift piles and proposed a calculation method for the ultimate bearing capacity of the bottom uplift pile after introducing the relative flexibility parameter of the pile. Pan^13^ discussed the analytical calculation method of the ultimate bearing capacity of a bottom uplift pile in a single sandy soil layer under ideal conditions and gave the calculation formula of the bearing capacity of an uplift pile with a bottom in a composite soil layer. Nie et al.^14^ found that the shear softening characteristics of the soil around the pile should be taken into account in calculating the ultimate bearing capacity of an uplift pile in a dense sand foundation. The shear stress of pile soil at different depths cannot reach the ultimate strength synchronously, and the value of the ultimate bearing capacity is lower than that of the traditional theoretical calculation. Zhao^15^ analysed the influence of the Poisson effect on the side resistance of a bottom uplift pile and the influence of different locations of uplift force on the distribution of the pile side resistance. Song^16^ found that the stress state of the soil mass was the fundamental reason for the change in friction, which not only affects the value of pile‒soil interface strength but also makes the soil mass show different shear resistance under certain strength conditions. Bao et al.^17^ found that the distribution form of the axial force on the pile shaft of the bottom uplift pile in silt is related to the loading position and that the maximum value of the friction resistance of the top pressure pile and the bottom uplift pile occurs at the 1/3 pile length from the pile loading point.

As mentioned above, scholars have conducted in-depth analyses on normal uplift piles and bottom uplift piles from the aspects of bearing capacity, friction and axial force of the pile shaft through indoor tests, numerical simulation and theoretical methods. Additionally, these researchers have studied the influence of soil layer parameters and the Poisson effect on the ultimate bearing capacity and have provided the corresponding formula for calculating the ultimate uplift bearing capacity. However, current studies on both normal uplift and bottom uplift piles have mainly been carried out for single soil layers, and the research on the bearing characteristics in a layered foundation is less. Furthermore, there have been few comparative studies on two kinds of uplift piles in the same soil layer. In this paper, based on the pile foundation test of the Lanzhou Zhongchuan International Airport Phase III Expansion Project, the upper pile of the pile foundation self-balanced test method was regarded as the bottom uplift pile, and the anchor pile in the anchor pile method was regarded as the normal uplift pile. On the basis of using field tests to verify the correctness of the numerical model, the load displacement relationship and the variation law of the axial force and friction of the normal uplift pile and bottom uplift pile in the layered foundation of Lanzhou Xinqu were explored in this paper. The difference in the ultimate bearing capacity and axial force and friction between the bottom uplift pile and normal uplift pile under the same conditions was analysed, and a comparison between the characteristics and advantages of the bottom uplift pile and the normal uplift pile was revealed. This paper could provide a reference for the popularization of bottom uplift piles in practical projects.

## Engineering overview

### Engineering background

The Lanzhou Zhongchuan International Airport Phase III Expansion Project is located in northern Lanzhou city, approximately 67 km from the downtown highway, and is an important part of the comprehensive transportation system of Gansu Province. Pile foundations with diameters ranging from 0.8 to 1.5 m and pile lengths ranging from 15 to 50 m are used for the T3 terminal, GTC traffic centre, landing platform viaduct and auxiliary buildings. The buried depth of the pile foundation includes plain silt, fine sand, breccia, silt and other multilayer soil, and the engineering properties of each soil layer are quite different. In this paper, five test piles with similar soil conditions in the GTC traffic centre were selected for analysis.

### Engineering geological conditions

According to the regional geological data and drilling exposure, the stratum structure in the test site is relatively complex. The stratigraphic structure is typical of the alluvial and proluvial origin of the Qinwangchuan Basin. The regularity in the horizontal and vertical directions and the sequence continuity are poor. The main soil layers and relevant parameters within the scope of the pile foundation test at the site are shown in Table 1.

**Table 1:** Soil parameters.

Geotechnical type	Depth (m)	Unit weight of soil (kN/m^3^)	Poisson's ratio	Cohesion (kPa)	Internal friction angle (°)
Plain fill	0–2	15.72	0.17	24.3	23.4
Fine sand	2–6	16.80	0.32	–	33.0
Breccia	6–16	21.30	0.18	–	40.0
Silt	16–20	17.44	0.25	22.8	23.9
Silty clay	20–30	19.21	0.33	22.6	21.2

## Static load test of a single pile on site

### Introduction and selection of test piles

The bearing capacity of the pile foundation were tested by the self-balancing method and anchor pile method. In the self-balancing method, the bottom load box of the upper section of the pile caused it to undergo an upwards displacement. The mode of the bottom stress was equivalent to the mode of the bottom uplift pile. The field test results could be used as the basis for the analysis of the bottom uplift pile^18^. In the anchor pile method test, the anchor pile was selected as the research object, and the force on its top was first stressed and then transmitted to the whole pile body, which was the force bearing mode of an ordinary uplift pile. The field test data could be used as the analysis basis of ordinary uplift piles.

Three test piles (#12, #13 and #14) from the GTC traffic centre were selected for the self-balancing test. The pile diameter is 0.8 m, the pile length is 30 m, and the pile length above the load box is 22.5 m. The maximum loading value of the test pile was 8500 kN, but the three upper piles had not reached the failure state. Two test anchor piles in the GTC traffic centre were selected as the research object. The diameter of the anchor pile is 0.8 m, and the pile length is 22.5 m (consistent with the upper section of the self-balanced test pile). When the maximum loading value reached 7000 kN, loading was stopped. Therefore, the uplift force borne by each anchor pile was 1750 kN (one pile with four anchors), which also did not reach the failure state, and the vertical displacement was small.

### Test method

**Figure 2:**
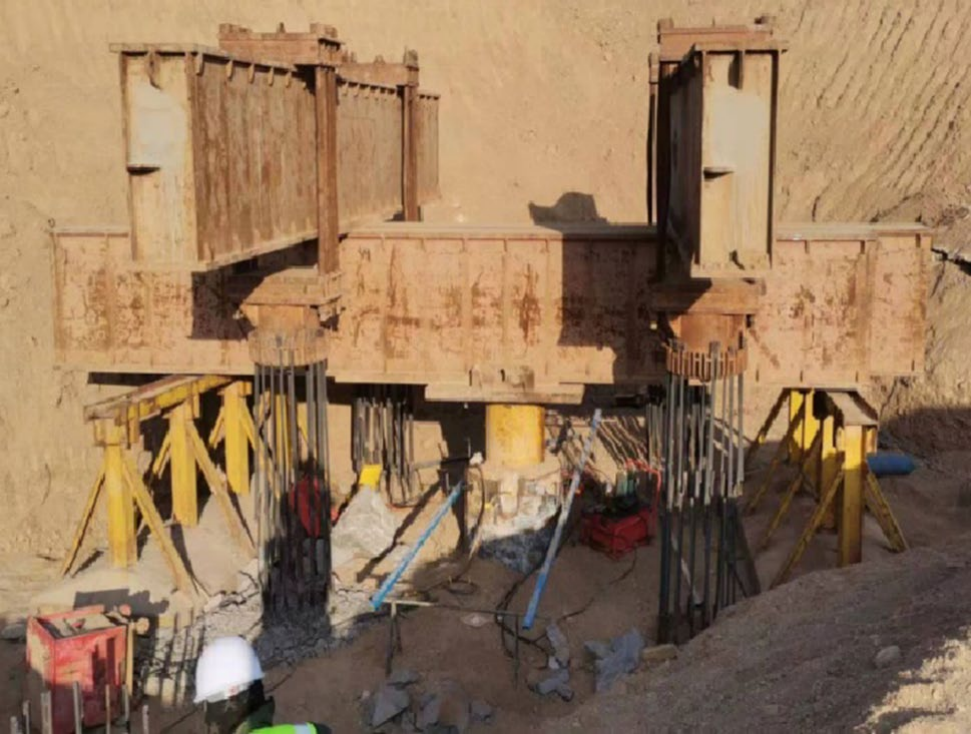
Test drawing of the anchor pile method.

An anchor pile reaction beam device and a QF-1000T-20b hydraulic jack were used for the anchor pile method test. The distance between the pile top settlement measurement plane and the pile top was more than 40 cm. Four displacement sensors were installed at the four endpoints of the two orthogonal diameters of the settlement measurement plane. One compressive pile used four anchor piles, which were loaded by jacks between the reaction beams and compressive piles. Figure 2 exhibits the test loading diagram.

In the self-balancing method, a test pile was divided into two parts: the upper pile and the lower pile. The upper and lower piles were connected by a load box. After the pile sunk, an upwards force was applied to the upper pile and a downwards force was applied to the lower pile through the load box (the two forces were equal in size and opposite in direction).

In this process, the upper pile was only subjected to the upwards force exerted by the load box and would also produce upwards displacement under the action of the load box. Therefore, the upper pile could be equivalent to the bearing form of the bottom uplift pile.

**Figure 3:**
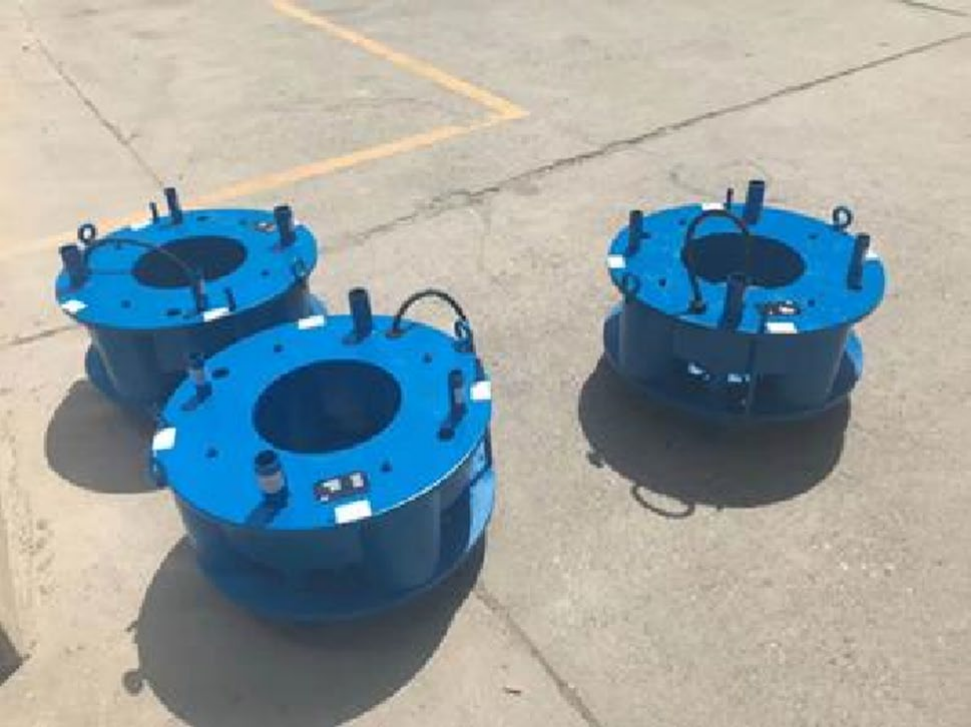
Load box.

**Figure 4:**
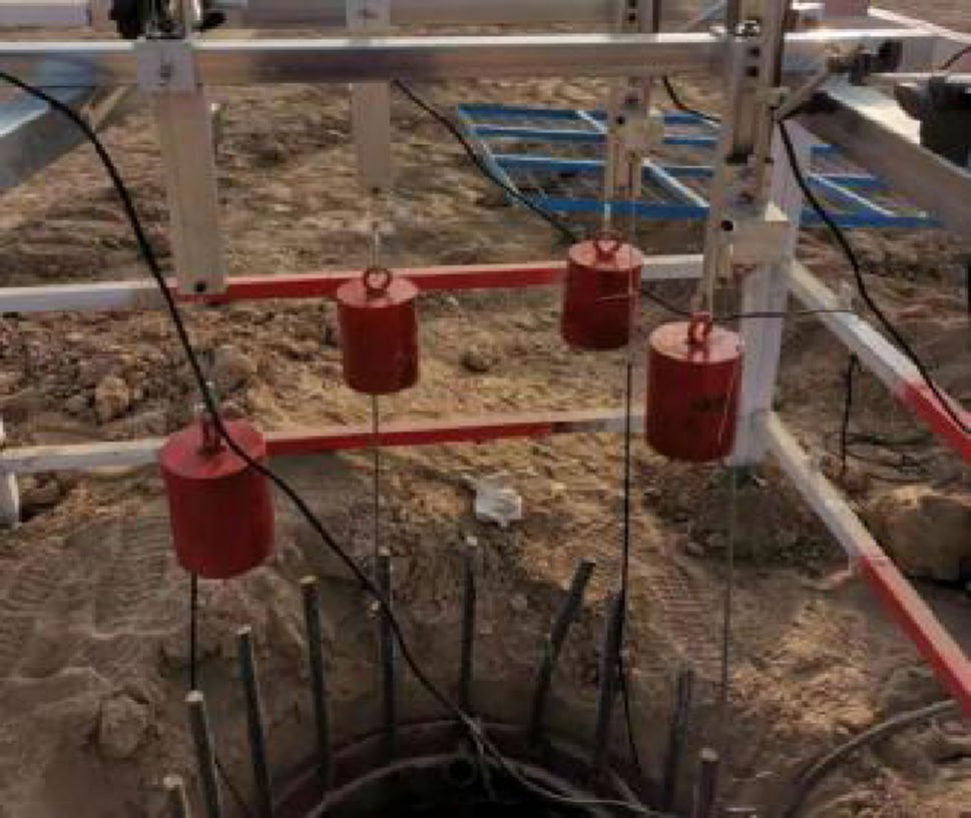
Displacement measuring device.

The outer diameter of the annular load box tested by the self-balancing method was 720 mm, the inner diameter was 380 mm, the maximum loading value was 17,000 kN, and the stroke was 100 mm. Figure 3 shows the load box. The electronic displacement sensor used to monitor the displacement had a measuring range of 50 mm. Four sensors were arranged for each pile and fixed on the benchmark steel beam through the magnetic meter base, as shown in Fig. 4.

The slow maintenance load method was adopted for the loading of two kinds of piles, and the loading was carried out step by step with equal amounts. During loading, the load transmission should be uniform, continuous and free from impact, and the variation range of each level of load during maintenance should not exceed ± 10% of the level of load.

Displacement observation: after each level of loading, the displacement was measured and read at 5 min, 15 min, 30 min, 45 min and 60 min in the first hour and then every 30 min. After reaching stability, the next level of load should be added.

### Test result

Table 2 shows the monitoring results of the vertical uplift displacement of the anchor pile in the anchor pile method test, and Table 3 shows the monitoring results of the upwards displacement of the upper pile in the self-balanced test method. It can be seen from the results that because the test equipment and loading conditions were the same and the soil conditions were basically similar, the vertical displacement obtained by each test method was basically the same under the same load. As the anchor pile method adopted one pile with four anchors, the load of the anchor pile was small when the loading was stopped (7000 kN). The vertical displacements of the two anchor piles under the action of the pile top pulling force of 1750 kN were only 1.87 mm and 2.07 mm, with an average of 1.97 mm. When the self-balanced upper section pile stops loading (8500 kN), the vertical displacements of the three piles were 12.24 mm, 13.60 mm and 11.55 mm, with an average of 12.46 mm. The specific analysis of the test results is carried out in the fourth section in combination with the finite element simulation results.

**Table 2:** Data sheet of the anchor pile method.

Number	Pile diameter (m)	Pile length (m)	Load when stop loading (kN)	Uplift amount when loading is stopped (mm)	Average uplift (mm)	Destruction
MZ01	0.8	22.5	1750	1.87	1.97	Undamaged
MZ04	0.8	22.5		2.07		Undamaged

**Table 3:** Data sheet of the upper pile of the self-balancing method.

Number	Pile diameter (m)	Pile length (m)	Pile length of upper section (m)	Load when stop loading (kN)	Uplift amount when loading is stopped (mm)	Average uplift (mm)	Destruction
#12	800	30	22.5	8500	12.24	12.46	Undamaged
#13	800	30	22.5	8500	13.60		Undamaged
#14	800	30	22.5	8500	11.55		Undamaged

## Establishment of the finite element model

### Model establishment

ABAQUS finite element software was used to model and analyse the normal uplift pile and the bottom uplift pile. Since the uplift piles of the two kinds of bearing capacity field tests are located in the GTC traffic centre area and the soil layer parameters change little, the same soil layer parameters were set for the two types of uplift piles in the modelling process, and the specific soil layer parameters are shown in Table 1. The constitutive model of the soil layer was the Mohr–Coulomb model.

**Figure 5:**
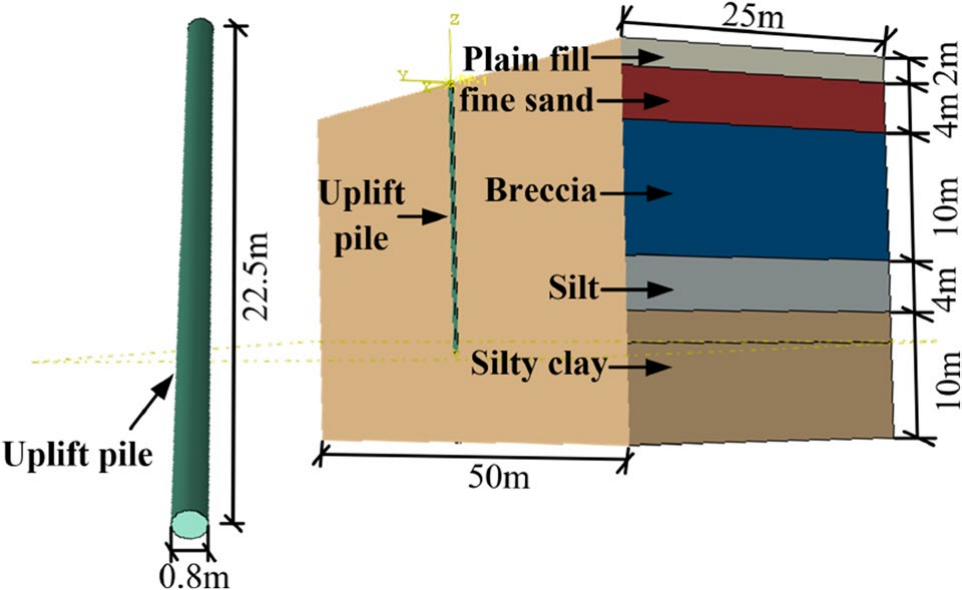
Structural model diagram.

According to the field test, the length of the normal uplift pile and the bottom uplift pile is 22.5 m, and the constitutive model of the two kinds of piles was a linear elastic model. To eliminate the influence of the boundary effect on the simulation results and consider the calculation efficiency, the length, width and height of the soil model were 50 m × 50 m × 30 m. The top surface of the soil model was a free boundary, the bottom surface was constrained by three sides, and the other four sides were normal constraints. The pile‒soil interface was simulated by a penalty function, and the friction coefficient was 0.5. The finite element model of the uplift pile is shown in Fig. 5. For a normal uplift pile, a uniform pulling force was applied on the pile top; for a bottom uplift pile, a uniform compressive stress was applied to the pile bottom.

### Determination basis of the ultimate bearing capacity of uplift pile

According to the existing research results, for the self-balancing test method, when the *Q-s* curve is slowly deformed, the load corresponding to its upwards displacement *s* = 40–60 mm can be taken as the ultimate bearing capacity^19^. Dong^20^ also suggested that the uplift force corresponding to the uplift amount of 30–40 mm should be used as the vertical uplift ultimate bearing capacity of the slowly deformed pile. According to the test results and simulation results, when the uplift displacement of the two kinds of uplift piles studied in this paper reached 40 mm, the pile and soil were not damaged, and the *Q-s* curve was still in the slowly rising stage, without abrupt change. Therefore, the bearing capacity corresponding to the uplift displacement of 40 mm at the pile top was taken as the judgement basis of the ultimate uplift bearing capacity of the two types of uplift piles.

### Calculation conditions

The finite element simulation calculation was divided into three parts: ① The correctness of the finite element model and the selected parameters were verified by field tests; ② tensile stress was applied on the top of the normal uplift pile until the displacement of the pile top reached 40 mm; and ③ compressive stress was applied to the pile bottom of the bottom uplift pile until the displacement of the pile top reached 40 mm.

## Result analysis

### Comparison and analysis of *Q-s* curves of two kinds of uplift piles

**Figure 6:**
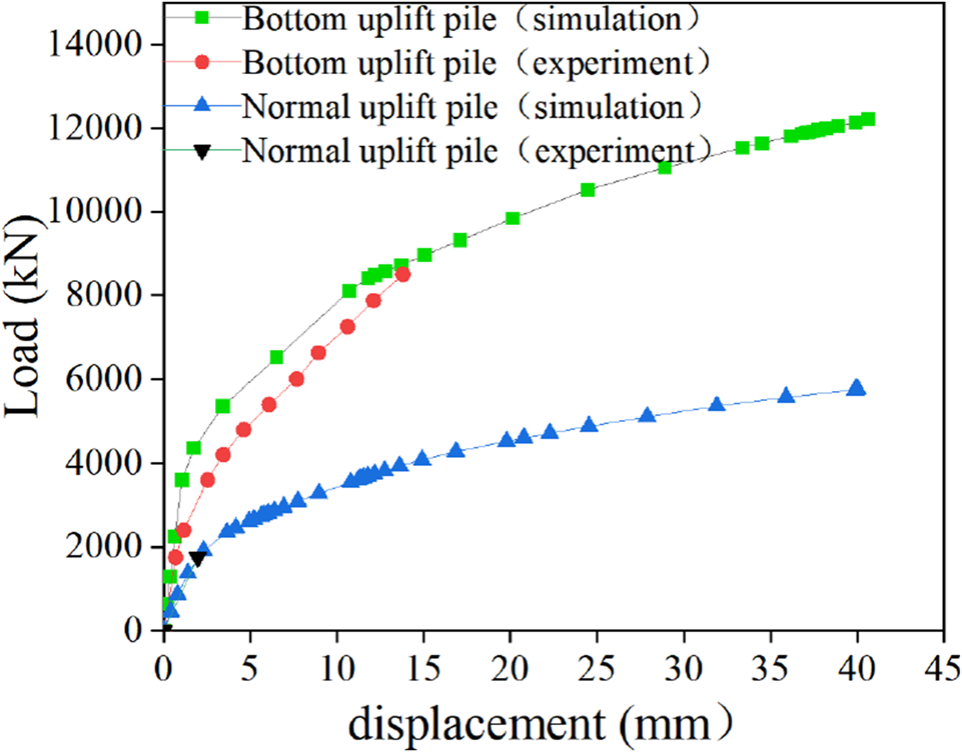
Q–s curves of the uplift pile.

To make sure the accuracy of the analysis results, the average displacement values of the upper sections of the #12, #13 and #14 self-balanced test piles were taken as the field monitoring results of the bottom uplift pile. The average displacement values of the MZ01 and MZ04 test piles were taken as the field monitoring results of the normal uplift pile. The *Q–s* curves of two kinds of uplift piles obtained from field tests and numerical simulation are shown in Fig. 6.

According to the *Q–s* curves obtained, it can be seen that under the action of 1750 kN, the field test displacement of the normal uplift pile is 1.97 mm, while the numerical simulation result is 2.02 mm, which is very close. The change rule of the field test curve and the numerical simulation curve of the bottom uplift pile is similar, and the simulation results are slightly larger than the measured results, but the load difference under the same displacement is not more than 10%. Therefore, the model established in this paper is reliable and can be used for further analysis of the bearing characteristics of normal uplift piles and bottom uplift piles.

Figure 6 shows that the *Q–s* curve of the normal uplift pile can be divided into two sections, with 1916 kN as the limit (also the proportional limit). When the load is within 1916 kN, the displacement will slowly increase linearly with the load, which is a linear elastic stage. After the load exceeds 1916 kN, although the displacement still increases linearly with the load, the growth rate increases significantly. When the load is 5762 kN, the pile top displacement reaches 40 mm, which is a plastic stage. Different from the normal uplift pile, the *Q–s* curve of the bottom uplift pile has three sections, and the boundaries of the three sections are 3605 kN (proportional limit) and 8730 kN. When the load is within 3605 kN, the displacement increases slowly, and the pile‒soil interface is in an elastic state. When the load is between 3605 and 8730 kN, the curve slope increases slightly, and this stage can be regarded as a transition section. The displacement growth rate increases rapidly until the load exceeds 8730 kN, and the pile top displacement reaches 40 mm at 12,209 kN.

By comparing the *Q-s* curves of the two types of uplift piles, it can be clearly seen that under the same soil layer conditions and the allowable pile top displacement, the ultimate bearing capacity of the normal uplift pile is only 5762 kN, while the ultimate uplift bearing capacity of the bottom uplift piles is 12,209 kN, 2.12 times that of the normal uplift pile. And, under the same load, the displacement of the pile top of the bottom uplift pile is also significantly smaller than that of the normal uplift pile. For example, under the limit load of 5762 kN for a normal uplift pile, the displacement of the pile top of the normal uplift pile reaches 40 mm, while the displacement of the pile top of the bottom uplift pile is less than 5 mm^21^.

The ultimate bearing capacity of the bottom uplift pile is better than that of the normal uplift pile, which is mainly related to their stress modes: (1) The normal uplift pile is pulled at the top of the pile, and the friction around the pile at the pile top comes into play first. However, the bottom uplift pile of the pile is pressed, and the friction of the soil around the pile bottom comes into play first. The size of the friction is proportional to the normal stress, and the self-weight stress of the upper soil on the soil at the bottom of the pile is significantly greater than the soil at the top of the pile. (2) Because of the Poisson effect of the pile body, when the normal uplift pile is tensioned at the top of the pile, its pile diameter decreases, and the pile‒soil interface presents a separation trend. However, when the pile bottom bears pressure, the pile diameter increases, the pile‒soil interface shows a tendency of compaction, and the friction is naturally greater than that of normal uplift piles. (3) Because the load of normal uplift pile acts on the pile top, the stretching amount of the pile body is fully included in the pile top displacement. Under the condition of limiting the pile top displacement, the displacement of the pile top of the normal uplift pile soon reaches the limit value, and the friction resistance of the soil around the pile cannot be fully exerted. However, the load bearing part of the bottom uplift pile is at the pile bottom, and the displacement of the pile will not be transmitted to the top until the pile body is completely compressed. This delays the development of the displacement of the pile top, thus giving full play to the friction.

### Comparative analysis of the axial force of the two kinds of uplift piles

**Figure 7:**
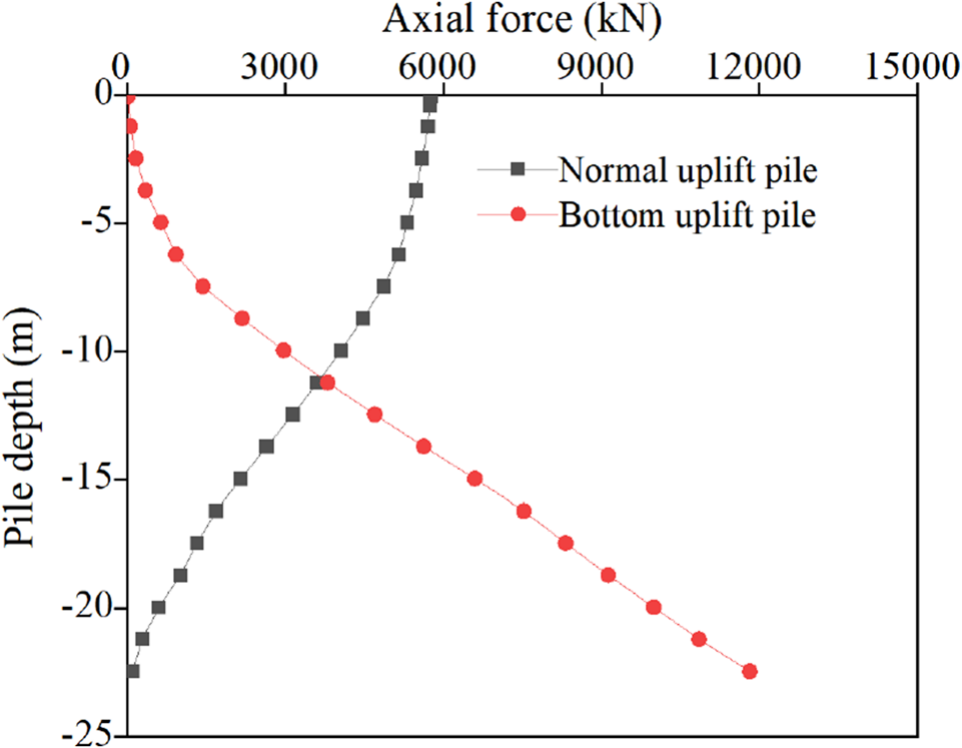
Axial force distribution of the uplift pile.

**Figure 8:**
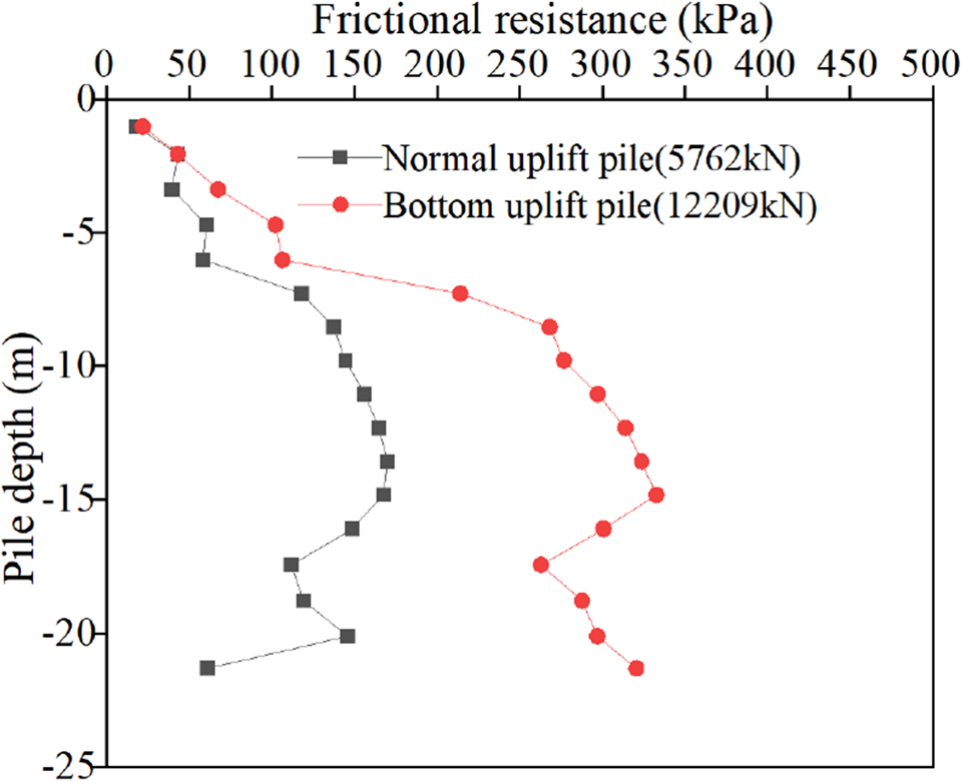
Ultimate frictional resistance of the uplift pile.

The distribution of the axial force and friction resistance of the two kinds of uplift piles under the action of ultimate bearing capacity along the pile shaft is shown in Figs. 7 and 8.

It can be seen from the axial force distribution diagram that the axial force distributions of the two types of uplift piles under load are opposite. The maximum axial force appears at the pile top, which decreases gradually with depth, and the axial force decreases to 0 kN when approaching the pile bottom. Instead, the maximum axial force of the bottom uplift pile appears at the pile bottom, which decreases gradually from the pile bottom to the pile top and reaches the minimum value of 0 kN at the pile top.

The smaller the slope of the axial force change curve is, the faster the load transfer, and vice versa. The axial force variation curve of the normal uplift pile has the largest slope within the range of 6 m at the top, indicating that the pile friction in this section is small. Additionally, the slope within the range of 6–16 m decreases, indicating that the pile friction in this section has increased, and the slope below 16 m shows an increasing trend. The axial force variation curve of the bottom uplift pile can be divided into two sections. Due to the Poisson effect in the depth range of 0–6 m, the friction is slightly greater than that of the normal uplift pile. The slope of the curve within the range of 6–22.5 m is significantly reduced (smaller than that of the normal uplift pile), indicating that the frictional resistance is increasing and significantly greater than that of the normal uplift pile.

A comparison of the soil layer around the pile (Table 1) indicates that the size of friction has a strong relationship with the nature of the soil layer. The area within 6–16 m is the breccia layer, which has the best soil properties, so its friction is the largest. In contrast, the area within 0–6 m is relatively poor for plain fill and gravel, and the area within 16–22.5 m is relatively poor for silt and silty clay, so the friction is relatively small, and obvious softening of friction occurs.

The distribution curve of friction along the pile shaft of two kinds of uplift piles under the action of ultimate bearing capacity verifies the correctness of the above analysis. It can be seen from Fig. 8 that due to the layered characteristics of the soil layer, the friction around the pile does not change linearly along the depth direction, and the jump at the soil layer boundary is particularly obvious. However, in the same layer of soil, the friction can change linearly along the depth. The frictional resistance of the normal uplift pile increases first and then decreases along the depth direction. The reason for the increase is that with increasing depth, the dead weight of the soil gradually increases, and the normal stress increases. The reason for the reduction is that the part of the drawing force transmitted to the bottom is small, resulting in the friction at the pile bottom not being fully exerted. The friction extension depth of the bottom uplift pile basically shows an increasing trend. The normal stress of the soil near the pile bottom on the pile body is large, so the friction is large. When the bottom uplift pile bears pressure at the pile bottom, the friction of the soil near the pile bottom is first exerted, and then as the load is transferred along the pile top, the friction of the middle and top of the pile body is gradually mobilized until the pile top. Under the same pile top displacement (40 mm), the maximum lateral friction of the normal uplift pile is only 150 kPa, while that of the bottom uplift pile is close to 350 kPa.

### Analysis of the friction resistance of the two kinds of uplift piles under different loads

**Figure 9:**
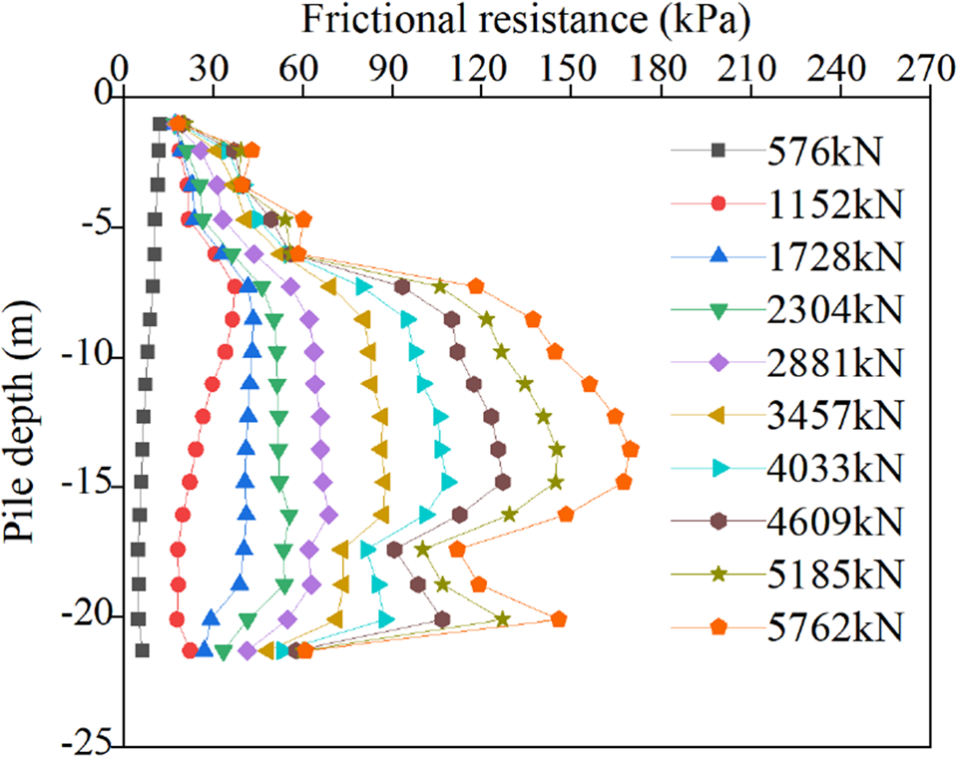
Friction resistance of the normal uplift pile.

**Figure 10:**
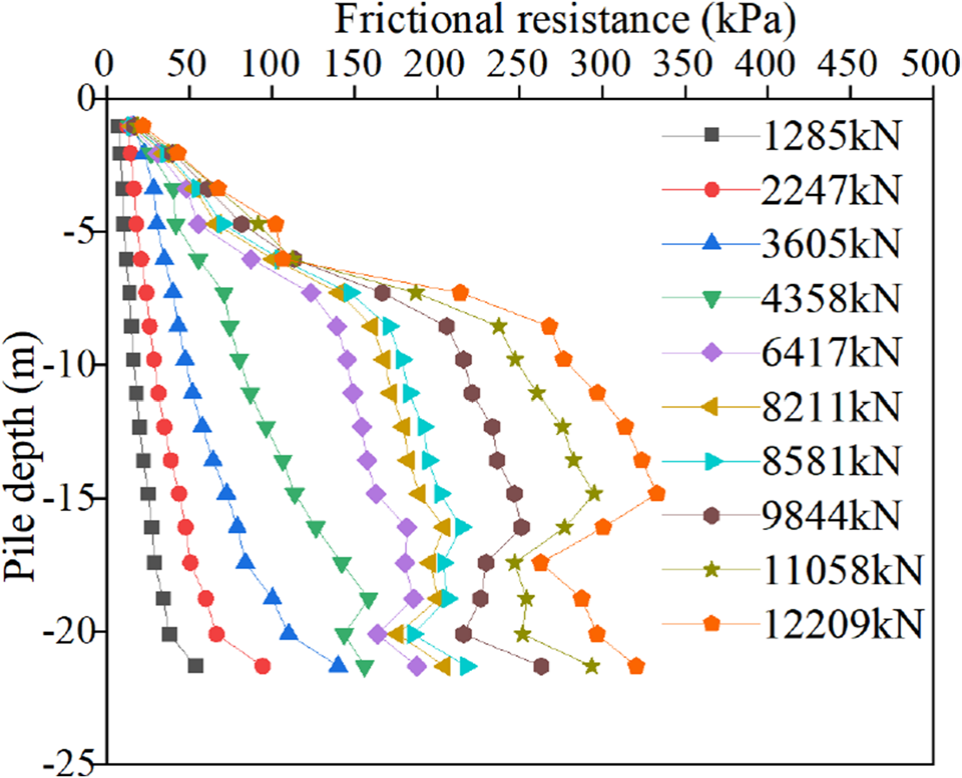
Friction resistance of the bottom uplift pile.

As the main component of the uplift bearing capacity, the process of pile shaft friction is very important for the study of the ultimate uplift bearing capacity. In this paper, the ultimate bearing capacity calculated by ABAQUS software is divided into 10 levels, and the pile shaft friction data under the 10 level loads of the two kinds of uplift piles are extracted, as shown in Figs. 9 and 10.

Figure 9 shows that at the initial stage of loading (0–2304 kN), with increasing load, the friction force develops gradually from the top to the bottom. Due to the poor nature of the soil near the pile top and the small self-weight pressure of the upper soil, the friction force quickly approaches the extreme limit. At this stage, the friction between the pile and soil in the deep soil layer is relatively small and is not close to the limit friction, so the influence of the properties of the deep soil on the friction is not apparent, and the friction increases uniformly within the range of pile lengths below 6 m. When the load exceeds 2304 kN, the soil friction within the range of 16–22.5 m softens, and the increase in soil friction within the range of 6–16 m increases significantly. With a continuous increase in load, the influence of the soil layer properties on the exertion of friction becomes increasingly obvious. The friction of soil layers with good properties continues to increase, while the increase in friction of soil layers with poor properties is relatively slow. As the normal uplift pile is tensioned at the top of the pile, most of the load is resisted by the friction of the upper and middle soil mass, and the load transmitted to the pile bottom is limited, so the friction of the soil layer near the pile bottom is always small. In the limit state, the maximum value of friction resistance appears at approximately 2/3 of the pile length below the pile top, and most of the pullout load is shared by the middle soil layer.

Figure 10 shows that the skin friction of the bottom uplift pile is completely opposite to that of the normal uplift pile. As the pile bottom bears the pressure, the skin friction of the soil near the pile bottom first plays and gradually develops to the pile top with increasing load. When the uplift load is within 3605 kN, the law of the friction distribution curve is similar, which is the maximum at the pile bottom. With a reduction in the pile depth, the friction decreases and reaches the minimum at the pile top. When the uplift load reaches 4358 kN, the friction resistance of silt and silty clay within the range of 16–22.5 m softens. With increasing load, the increase in friction resistance in this section slows down significantly, and more load is borne by the breccia layer within the range of 6–16 m. The location of the maximum friction also changes gradually. Similar to normal uplift piles, the maximum friction still appears approximately 2/3 of the pile length below the pile top in the limit state. As the pile bottom is loaded, the load is difficult to transfer to the pile top, so the friction of the soil near the pile top is always small.

A comparison of the friction distribution curves of the two types of uplift piles indicates the following: (1) the bottom uplift pile can make full use of the characteristics of the soil layer at the bottom of the pile that bears the heavy weight of the upper soil mass and can provide greater lateral resistance so that the lateral friction of the soil mass at the bottom of the pile can be exerted first. Moreover, it can also overcome the shortcomings of the small lateral friction of the soil mass near the top of the pile. (2) The soil layer conditions have different effects on the bearing characteristics of the two types of uplift piles. For normal uplift piles, the side friction of the soil layer near the pile top is small, and the load cannot be effectively transmitted to the pile bottom, so the middle of the pile shaft is the main part to resist the uplift load. However, the background project of this paper just happens to have good soil properties in the middle of the pile shaft, so it has little impact on the bearing characteristics of normal uplift piles, and the distribution curve of friction along the pile length is still "parabolic"^18^. For the bottom uplift pile, the middle and lower parts of the pile shaft are the main parts to resist the pullout force, while the soil layer near the pile bottom in the background project is relatively poor, so the distribution curve of the friction along the pile length changes from a "straight line" type to a "double line" type^4^.

### Comparative analysis of the stage friction of two kinds of uplift piles

**Figure 11:**
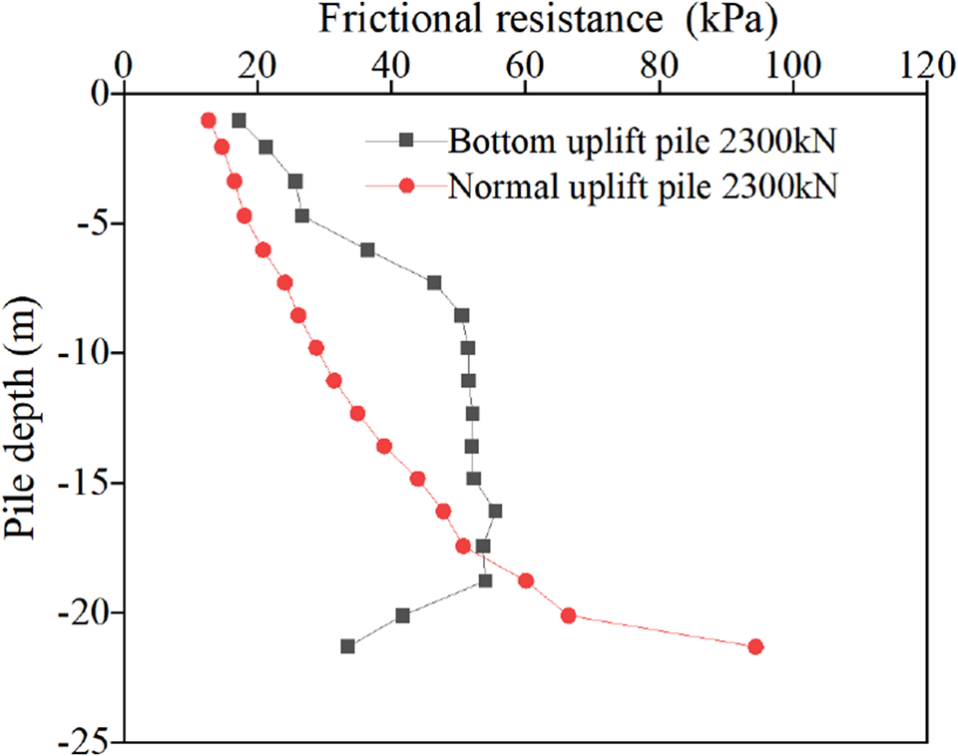
Friction resistance in the first stress stage.

**Figure 12:**
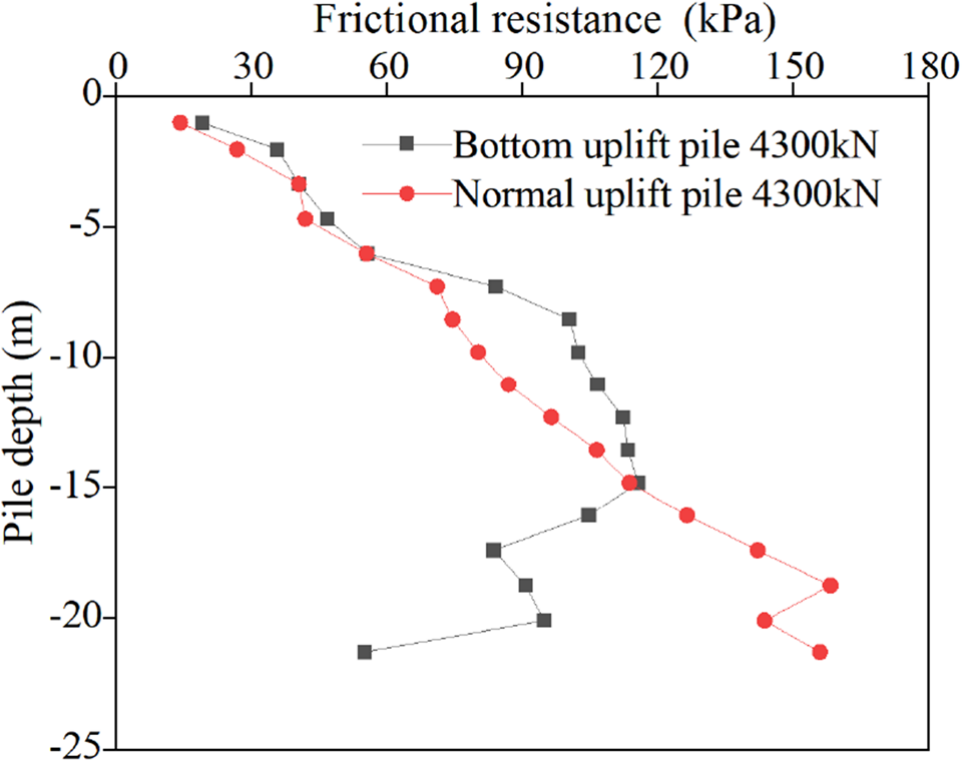
Friction resistance in the second stress stage.

**Figure 13:**
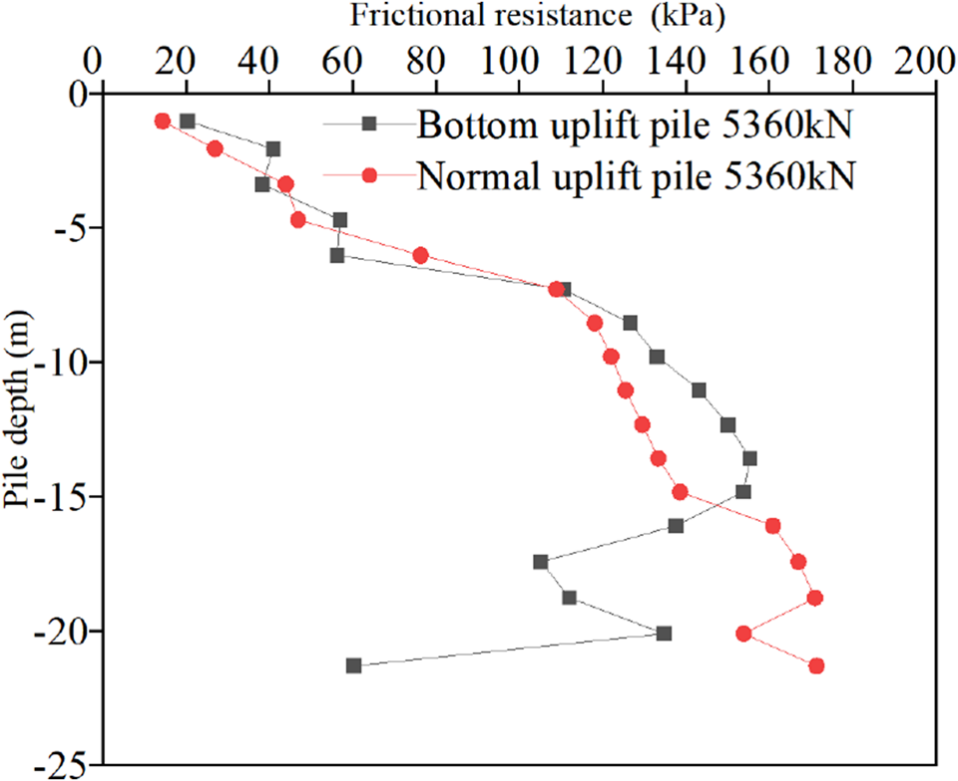
Friction resistance in the third stress stage.

To further intuitively compare and analyse the relevant laws of the friction resistance of the two types of uplift piles, the friction resistance curves of the two types of uplift piles under the action of three levels of the same load (2300 kN, 4300 kN and 5360 kN) are taken for comparative analysis, as shown in Figs. 11, 12 and 13.

Because of the difference in the ultimate bearing capacity, the change curves of the friction resistance of the normal uplift pile and uplift pile with the base under the same load show different characteristics.

For normal uplift piles, the ultimate bearing capacity is only 5762 kN, 2300 kN is approximately 40% of the ultimate bearing capacity, 4300 kN is 75%, and 5360 kN is close to 95%. Under the third-order load, the distribution curve of friction along the depth of the normal uplift pile exhibits a parabolic shape. From the change in the friction curve, under a relatively small load, the soil near the pile bottom also has a certain friction, and it is not until the soil friction at the pile top reaches the limit that the load is transmitted downwards. The exertion degree of friction has a strong relationship with the relative displacement between the pile and soil, and the exertion degree of friction increases with increasing relative displacement between the pile and soil.

For the bottom uplift pile, its ultimate bearing capacity is 12,209 kN, 2300 kN corresponds to 20% of the ultimate bearing capacity, 4300 kN is 35%, and 5360 kN is only 44%. Under the action of 2300 kN and 4300 kN loads, the distribution curve of the friction resistance along the depth of the bottom uplift pile can also be approximately regarded as a straight line. When the load reaches 5360 kN, the curve shows obvious "broken line" characteristics. Since the load is relatively small, the friction resistance of the soil at the pile bottom does not reach the limit value. When the load reaches approximately half of the limit value, the soil at the pile bottom is still the main force resisting the load.

A comparison of the friction curves of two types of uplift piles under the same level of load shows that, due to different loading modes, under the same load, normal uplift piles must give full play to the friction of the upper and middle soil to make up for the shortcomings of the lower soil friction. Relatively speaking, the bottom uplift pile accumulates more reserves, which is also why the ultimate bearing capacity of the bottom uplift pile is greater. It is worth noting that in the engineering background of this paper, the soil layer near the pile bottom is relatively poor, which affects the bearing characteristics of the bottom uplift pile. It can be inferred that, for example, under the condition of a uniform soil layer or good soil properties at the pile bottom, the ultimate bearing capacity of the bottom uplift pile is more than 3 times that of the normal uplift pile.

## Parameter analysis

On the basis of the bottom uplift pile model built in section "Establishment of the finite element model", to study the influence of various factors on the bearing capacity of the bottom uplift pile, in this section, different finite element software calculation models were built by changing different parameters of the bottom uplift pile (including the pile length, pile diameter and elastic modulus of the pile), as listed in Table 4.

**Table 4:** Calculation conditions.

Influence factors	Pile length (m)	Pile diameter (mm)	Elastic modulus of pile (N/mm^2^)
Pile length	24.5	800	3.45 × 10^4^
	22.5	800	3.45 × 10^4^
	20.5	800	3.45 × 10^4^
Pile diameter	22.5	700	3.45 × 10^4^
	22.5	800	3.45 × 10^4^
	22.5	900	3.45 × 10^4^
Elastic modulus of pile	22.5	800	3.35 × 10^4^
	22.5	800	3.45 × 10^4^
	22.5	800	3.55 × 10^4^

### Influence of pile length on the ultimate uplift bearing capacity of bottom uplift pile

**Figure 14:**
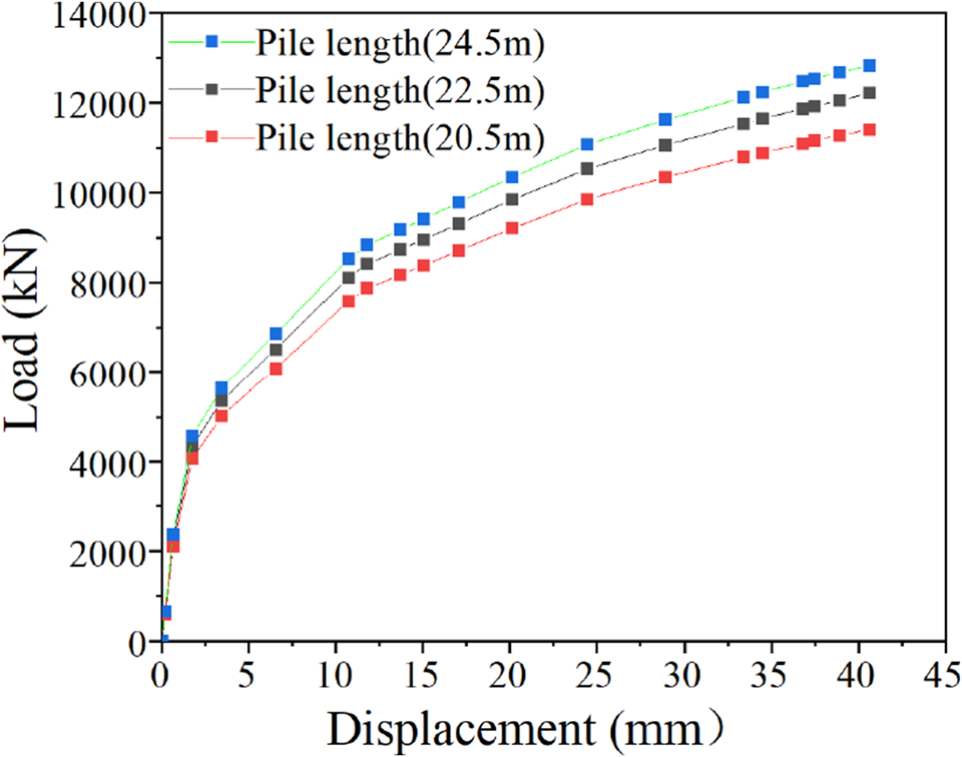
Influence of the pile length.

On the basis of the original finite element model with a pile length of 22.5 m, the other parameters remain unchanged, and the pile length of the model was changed to 20.5 m and 24.5 m. The ultimate bearing capacity of the bottom uplift pile with different pile lengths was calculated, and the *Q-s* curve is shown in Fig. 14.

Figure 14 shows that with increasing pile length of the bottom uplift pile, the ultimate uplift bearing capacity tends to increase under the same uplift displacement. The ultimate uplift bearing capacity corresponding to the original pile length of 22.5 m is 12,209 kN. When the pile length decreases by 2 m to 20.5 m, the ultimate uplift bearing capacity decreases to 11,410 kN, a decrease of 6.5%. When the pile length increases by 2 m to 24.5 m, the ultimate uplift bearing capacity increases to 12,830 kN, with an increase of 5.1%. When the displacement reaches 10.73 mm (uplift load is 8110 kN), the increase in uplift load slows down, and the increase in displacement increases. After that, the load reaches the limit load with a slower growth rate, and the displacement reaches the allowable displacement of uplift of 40 mm with a faster growth rate. The reason for this phenomenon is that with increasing pile length, the contact area between the pile and soil and the total side friction increase, which leads to the growth of the ultimate uplift bearing capacity of the bottom uplift pile.

### Influence of pile diameter on the ultimate uplift bearing capacity of bottom uplift pile

**Figure 15:**
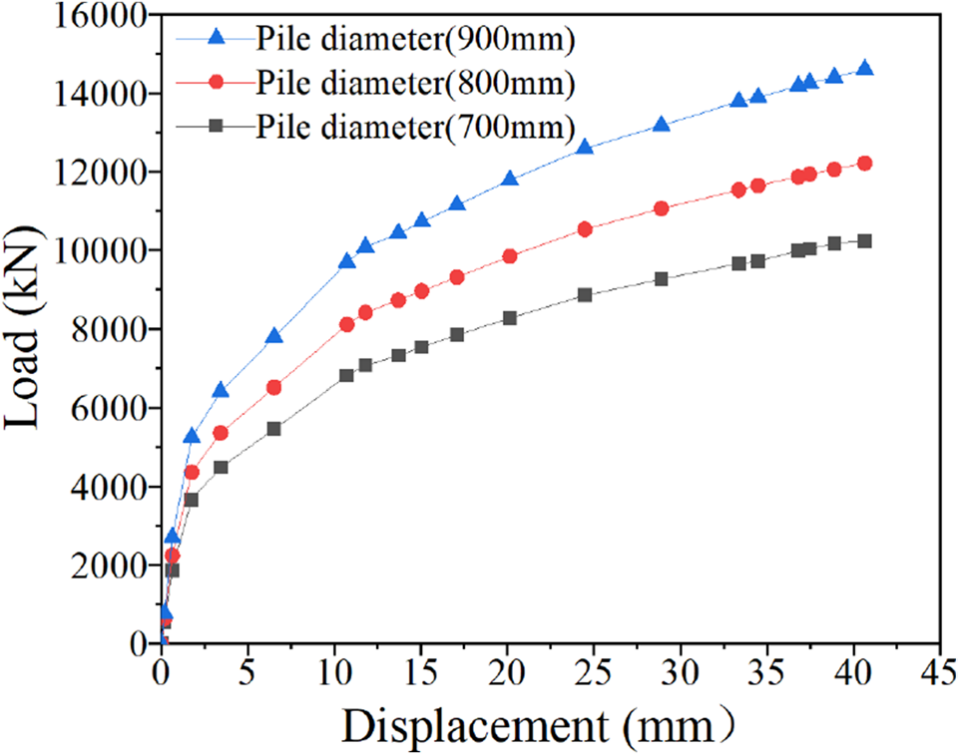
Influence of the pile diameter.

Taking the 800 mm pile diameter of the original model as the reference object, the pile diameter was changed to 700 mm and 900 mm, and the calculated ultimate uplift bearing capacity of the bottom uplift pile is shown in Fig. 15.

Figure 15 shows that the change in pile diameter has a great impact on the ultimate uplift bearing capacity of the bottom uplift pile. When the pile diameter increases from 700 to 800 mm and then from 800 to 900 mm, the ultimate uplift bearing capacity increases from 10,243 kN to 12,209 kN and then from 12,209 kN to 14,596 kN, with increases of 19.19% and 19.55%, respectively. The pile diameter can not only increase the contact area between the pile and soil, but also greatly increase the weight of the pile, which can play a positive role in improving the uplift bearing capacity. Compared with the change in the uplift bearing capacity caused by the change in pile length, the increase range of the uplift bearing capacity caused by the change in pile diameter is more significant.

### Influence of the elastic modulus of the pile on the ultimate uplift bearing capacity of the bottom uplift pile

**Figure 16:**
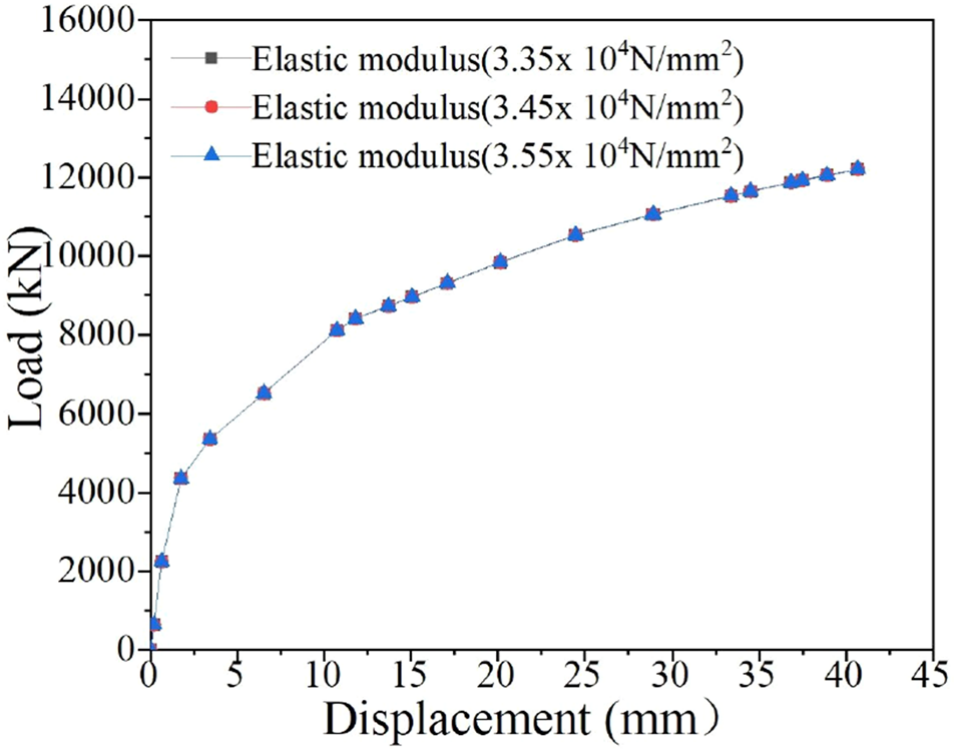
Influence of the elastic modulus of the pile.

The actual pile in the case of this paper was made of C50 concrete, with an elastic modulus of 3.45 × 10^4^ N/mm^2^. The elastic modulus of the pile will affect the deformation of the pile and further affect the ultimate uplift bearing capacity under the same pile top displacement (40 mm). To study the influence of the elastic modulus of concrete on the bearing capacity of piles, the concrete was changed into C45 and C55 concrete, with elastic moduli of 3.35 × 10^4^ N/mm^2^ and 3.55 × 10^4^ N/mm^2^, respectively. The *Q-s* curves for piles with different elastic moduli are shown in Fig. 16.

It is obvious from Fig. 16 that the elastic modulus of the pile has little influence on the ultimate uplift bearing capacity of the bottom uplift pile. When the elastic modulus changes from 3.45 × 10^4^ to 3.35 × 10^4^ N/mm^2^ and 3.55 × 10^4^ N/mm^2^, the ultimate bearing capacity of the uplift pile is almost unchanged. The reason for the above phenomenon is that the change in the elastic modulus of the pile is relatively small, which has little impact on the compression deformation of the pile, thus resulting in the constant ultimate uplift bearing capacity of the pile.

## Conclusion

In this paper, the bearing characteristics of bottom uplift piles in layered foundations were studied by using field tests and numerical simulation methods and were compared with those of normal uplift piles. The conclusions are as follows:

1. The uplift bearing capacity of the bottom uplift pile is much better than that of the normal uplift pile under the same displacement of the pile top. The *Q–s* curve of the normal uplift pile can be divided into an elastic stage and a plastic stage, while the *Q–s* curve of the bottom uplift pile can be divided into three sections, with more transition sections.
2. Under the same displacement of the pile top, the ultimate uplift bearing capacity of the bottom uplift pile can be more than twice that of the normal uplift pile. The reason for this phenomenon is that the soil friction at the pile bottom of the bottom uplift pile can be fully exerted, the Poisson effect increases the pile side friction, and the pile displacement can only be transmitted to the top after the pile is completely compressed.
3. The maximum axial force of the normal uplift pile appears at the pile top, which decreases gradually with depth, while the maximum axial force of the bottom uplift pile appears at the pile bottom, which decreases gradually from the pile bottom to the pile top.
4. The property and thickness of the soil layer have a great influence on the distribution curve of the friction resistance of the two kinds of uplift piles along the pile length. Under the soil layer condition of this project background, the friction distribution curve of the normal uplift pile is a "parabolic" type, while the friction distribution curve of the bottom uplift pile is a "double line" type. With changing soil layer conditions, the friction distribution curve may also change.
5. Soil properties play a decisive role in the change in friction. Although the exertion of friction is related to the buried depth of the soil, the soil layer with poor properties demonstrates an obvious friction softening phenomenon.
6. For the case of this paper, the influence of the pile diameter on the ultimate uplift bearing capacity is greater than that of the pile length, while the elastic modulus of the pile has little influence. The bearing capacity increases by 5.1% when the pile length increases to 24.5 m and decreases by 6.5% when the pile length decreases to 20.5 m. The bearing capacity increases by 19.6% when the pile diameter increases by 100 mm and decreases by 16.1% when the pile length decreases by 100 mm.

## Data Availability

The datasets generated during the current study are not publicly available because the engineering on which the test is based is not yet completed and the data is still confidential but are available from the corresponding author on reasonable request.
